# Supplementary material for: Growth exponents reflect evolutionary processes and treatment response in brain metastases
Source: NPJ Syst Biol Appl. 2023 Jul 21;9:35. doi: 10.1038/s41540-023-00298-1 (PMC10361973; doi:10.1038/s41540-023-00298-1)
Supplement: Supplementary file 1 — Reporting summary [file 41540_2023_298_MOESM1_ESM.pdf]

nature portfolio

Corresponding author(s): Beatriz Ocaña-Tienda

Last updated by author(s): 2023/07/06

Reporting Summary

Nature Portfolio wishes to improve the reproducibility of the work that we publish. This form provides structure for consistency and transparency in reporting. For further information on Nature Portfolio policies, see our Editorial Policies and the Editorial Policy Checklist.

Statistics

For all statistical analyses, confirm that the following items are present in the figure legend, table legend, main text, or Methods section.

n/a	Confirmed	
☐	☒	The exact sample size (*n*) for each experimental group/condition, given as a discrete number and unit of measurement
☐	☒	A statement on whether measurements were taken from distinct samples or whether the same sample was measured repeatedly
☐	☒	The statistical test(s) used AND whether they are one- or two-sided<br>*Only common tests should be described solely by name; describe more complex techniques in the Methods section.*
☐	☒	A description of all covariates tested
☐	☒	A description of any assumptions or corrections, such as tests of normality and adjustment for multiple comparisons
☐	☒	A full description of the statistical parameters including central tendency (e.g. means) or other basic estimates (e.g. regression coefficient) AND variation (e.g. standard deviation) or associated estimates of uncertainty (e.g. confidence intervals)
☐	☒	For null hypothesis testing, the test statistic (e.g. *F*, *t*, *r*) with confidence intervals, effect sizes, degrees of freedom and *P* value noted<br>*Give P values as exact values whenever suitable.*
☒	☐	For Bayesian analysis, information on the choice of priors and Markov chain Monte Carlo settings
☒	☐	For hierarchical and complex designs, identification of the appropriate level for tests and full reporting of outcomes
☒	☐	Estimates of effect sizes (e.g. Cohen's *d*, Pearson's *r*), indicating how they were calculated

Our web collection on statistics for biologists contains articles on many of the points above.

Software and code

Policy information about availability of computer code

Data collection	No software was used to collect the data
Data analysis	Matlab (2018b, 2022b), Julia 1.1.1, SPSS 24

For manuscripts utilizing custom algorithms or software that are central to the research but not yet described in published literature, software must be made available to editors and reviewers. We strongly encourage code deposition in a community repository (e.g. GitHub). See the Nature Portfolio guidelines for submitting code & software for further information.

Data

Policy information about availability of data

All manuscripts must include a data availability statement. This statement should provide the following information, where applicable:

- Accession codes, unique identifiers, or web links for publicly available datasets
- A description of any restrictions on data availability
- For clinical datasets or third party data, please ensure that the statement adheres to our policy

All data that support the plots within this paper and other findings of this study are available from the corresponding author upon reasonable request.

Research involving human participants, their data, or biological material

Policy information about studies with human participants or human data. See also policy information about sex, gender (identity/presentation), and sexual orientation and race, ethnicity and racism.

Reporting on sex and gender	Table 1 includes the number of patients in the study, detailling 38 men and 31 women.
Reporting on race, ethnicity, or other socially relevant groupings	Race, ethnicity or other socially groups were not had into account.
Population characteristics	The median age was 60 (33-78), other data related to the particular brain metastases are included in Table 1.
Recruitment	Patients included were all participants in the study MetMath (Metastasis and Mathematics), a retrospective, multicenter, nonrandomized study approved by five hospitals. All patients were diagnosed with BM in the period 2007-2021 and followed up with MRI according to standard clinical practice.
Ethics oversight	The study was approved by the corresponding institutional review boards: Fundación Instituto Valenciano de Oncología, Hospital Universitario HM Sanchinarro, Hospital Regional Universitario de Málaga, MD Anderson Cancer Center and Hospital Universitario de Salamanca.

Note that full information on the approval of the study protocol must also be provided in the manuscript.

Field-specific reporting

Please select the one below that is the best fit for your research. If you are not sure, read the appropriate sections before making your selection.

☒ Life sciences ☐ Behavioural & social sciences ☐ Ecological, evolutionary & environmental sciences

For a reference copy of the document with all sections, see nature.com/documents/nr-reporting-summary-flat.pdf

Life sciences study design

All studies must disclose on these points even when the disclosure is negative.

Sample size	The dataset includes 69 patients, accounting for 96 brain metastases.
Data exclusions	Brain metastases not fulfilling the inclusion criteria were excluded. Inclusion criteria were: First of all a minimum of three consecutive imaging studies, including a volumetric contrast-enhanced (CE) T1-weighted MRI sequence (slice thickness $\leq$ 2.00 mm, no gap) with no substantial imaging artifacts, at different time points, were required in order to allow for reliable lesion volume calculations. Secondly, an increase in tumor volume at each of the three time points was required, since it was desired to study the growth of either untreated or recurrent tumors. Next, only time points without previous SRS/WBRT treatments (for untreated cases) or with SRS/WBRT treatments received more than four months before the first imaging study were considered (treated cases), in order to exclude the potential confounding effect of acute inflammatory responses seen in some patients in the first MRI after SRS. Patients with prior surgical resection of the metastasis were excluded to avoid confounding effects, such as ischemia. Brain metastases lacking relevant clinical variables and/or data on treatments received, as well as those lacking consensus in segmentations (what may lead to uncertain values of the volumes) were also excluded.
Replication	Not applicable.
Randomization	Not applicable.
Blinding	Not applicable.

Reporting for specific materials, systems and methods

We require information from authors about some types of materials, experimental systems and methods used in many studies. Here, indicate whether each material, system or method listed is relevant to your study. If you are not sure if a list item applies to your research, read the appropriate section before selecting a response.

Materials & experimental systems

n/a	Involved in the study
☒	☐ Antibodies
☒	☐ Eukaryotic cell lines
☒	☐ Palaeontology and archaeology
☐	☒ Animals and other organisms
☐	☒ Clinical data
☒	☐ Dual use research of concern
☒	☐ Plants

Methods

n/a	Involved in the study
☒	☐ ChIP-seq
☒	☐ Flow cytometry
☐	☒ MRI-based neuroimaging

Animals and other research organisms

Policy information about studies involving animals; ARRIVE guidelines recommended for reporting animal research, and Sex and Gender in Research

Laboratory animals	Two animal models were used: 1) 17 female (2-3 months old) Foxn1nu mice for glioma xenographs and 2) 37 Athymic nu/nu (Harlan) mice of 4-8 weeks of age for brain metastasis
Wild animals	Not applicable.
Reporting on sex	Not applicable.
Field-collected samples	Not applicable.
Ethics oversight	Animal care and experimental procedures were performed in accordance to the European Union and National guidelines for the use of animals in research and were reviewed and approved by the Research Ethics and Animal Welfare Committee: for animal model 1) at our institution (Instituto de Salud Carlos III, Madrid) (PROEX 244/14); for animal model 2) approved at CNIO (PROEX 211/17)

Note that full information on the approval of the study protocol must also be provided in the manuscript.

Clinical data

Policy information about clinical studies
All manuscripts should comply with the ICMJE guidelines for publication of clinical research and a completed CONSORT checklist must be included with all submissions.

Clinical trial registration	Data used in this study did not belong to any clinical trial.
Study protocol	MetMath
Data collection	MetMath studiy was collected between 2007 and 2019.
Outcomes	Not applicable.

Magnetic resonance imaging

Experimental design

Design type	Not applicable.
Design specifications	Not applicable.
Behavioral performance measures	Not applicable.

Acquisition

Imaging type(s)	T1+Gd sequences.
Field strength	1, 1.5 and 3 T.
Sequence & imaging parameters	The volumetric contrast-enhanced T1-weighted MR imaging sequence used to delineate the BMs and compute their volumes was gradient echo using 3D spoiled gradient-recalled echo or 3D fast-field echo after intravenous administration of a single dose of gadobenate dimeglumine (0.10 mmol/kg) with a 6-to 8-minute delay. Imaging parameters were no gap, slice thickness of 0.52-2.0 mm (mean 1.3 mm), 0.4-1.1 mm (mean 0.5 mm) pixel-spacing and 0.4-2.0 mm sacing between slices (mean 1.0 mm).
Area of acquisition	Whole brain scan.

Diffusion MRI ☐ Used ☒ Not used

Preprocessing

Preprocessing software	Not applicable.
Normalization	Not applicable.
Normalization template	Not applicable.
Noise and artifact removal	Not applicable.
Volume censoring	Not applicable.

Statistical modeling & inference

Model type and settings	Not applicable.
Effect(s) tested	Not applicable.

Specify type of analysis: ☐ Whole brain ☒ ROI-based ☐ Both

Anatomical location(s): Brain.

Statistic type for inference (See Eklund et al. 2016)	Not applicable.
Correction	Not applicable.

Models & analysis

n/a	Involved in the study
☒	☐ Functional and/or effective connectivity
☒	☐ Graph analysis
☒	☐ Multivariate modeling or predictive analysis
